# Thermal Stability, Durability, and Service Life Estimation of Woven Flax-Carbon Hybrid Polyamide Biocomposites

**DOI:** 10.3390/ma17092020

**Published:** 2024-04-26

**Authors:** Mohsen Bahrami, Juana Abenojar, Gladis M. Aparicio, Miguel Angel Martínez

**Affiliations:** 1 IAAB, Materials Science and Engineering Department, University Carlos III of Madrid, 28911 Leganes, Spain; mamc@ing.uc3m.es; 2 Mechanical Engineering Department, University Pontificia Comillas of Madrid, 28015 Madrid, Spain; 3 Faculty of Basic Sciences, University Autónoma of Occidente, Cali 760030, Colombia; gmaparicio@uao.edu.co

**Keywords:** hybrid biocomposite, flax fibers, thermal properties, degradation, lifetime estimation

## Abstract

Woven flax-carbon hybrid polyamide biocomposites offer a blend of carbon fibers’ mechanical strength and flax’s environmental advantages, potentially developing material applications. This study investigated their thermal behavior, degradation kinetics, and durability to water uptake and relative humidity exposure and compared them with pure flax and carbon composites with the same matrix. The hybrid composite exhibited intermediate water/moisture absorption levels between pure flax and carbon composites, with 7.2% water absorption and 3.5% moisture absorption. It also displayed comparable thermal degradation resistance to the carbon composite, effectively maintaining its weight up to 300 °C. Further analysis revealed that the hybrid composite exhibited a decomposition energy of 268 kJ/mol, slightly lower than the carbon composite’s value of 288.5 kJ/mol, indicating similar thermal stability. Isothermal lifetime estimation, employing the activation energy (E_d_) and degree of conversion facilitated by the Model Free Kinetics method, indicated a 41% higher service life of the hybrid laminate at room temperature compared to the carbon laminate. These insights are crucial for understanding the industrial applications of these materials without compromising durability.

## 1. Introduction

In recent years, natural fiber composites have captured the spotlight across various industries, with applications ranging from automotive to aerospace, thanks to their renewability, lightness, and the potential they hold to replace conventional synthetic reinforcements [1]. Natural fibers like flax, hemp, and jute present an eco-conscious choice, valued for their ready availability, cost-effectiveness, and low environmental footprint [2]. The adoption of natural fibers within industries traditionally dependent on synthetic materials is increasing. This trend is driven by the fibers’ ability to enhance specific strength and stiffness, which are crucial attributes for achieving lightweight and durable components.

Among natural fibers, flax stands out for its impressive specific strength and modulus [3,4]. Flax fibers have a specific strength comparable to glass fibers and a specific modulus similar to that of carbon fibers, making them highly desirable for applications requiring lightweight and high-strength materials [5].

Compared to synthetic fibers, flax fibers have significant environmental advantages, including a reduced carbon footprint, enhanced end-of-life options, and less energy consumption during manufacture [3,6,7]. Studies have shown that natural fiber composites, such as those incorporating flax, have a significant potential to reduce environmental impacts in contrast to conventional materials. For instance, a particular study revealed that automotive parts based on flax require 83% less energy and are 40% more economical to produce in comparison to their counterparts made from glass fibers [8]. Furthermore, studies indicate that the substitution of glass fibers for flax fibers in fiber-reinforced polymer films may result in reductions in life cycle environmental impacts ranging from 20% to 50% across diverse impact categories [9].

Despite these advantageous properties, natural fiber composites are not without their challenges. Their high level of capillary water absorption (Table 1), exacerbated by waxes and atmospheric pollutants, leads to weak adhesion with polymer matrices, compounding their susceptibility to moisture-related issues [10,11,12]. On the thermal front, the intrinsic stability of natural fibers falls short of high-performance needs. The layered composition–cellulose, hemicellulose, and lignin–of these fibers decompose at varying temperatures, with complete disintegration occurring within the range of 300–450 °C [13,14]. To enhance thermal resistance, specific fiber constituents may need to be reduced, although this, paradoxically, could lower their thermal endurance and compromise the structural integrity of secondary cell walls [15].

To address these limitations, the hybridization approach emerges as a potent solution [26,27,28,29]. By combining the strengths of synthetic fibers like carbon fibers–known for their superior strength and thermal endurance–with the sustainable profile of natural fibers like flax fibers, the resultant composites not only enhanced mechanical properties [30,31,32,33,34] and thermal stability [32,35] but also improved moisture resistance [36,37], tailoring them more closely to the demands of high-performance applications.

The hybrid composites’ thermal behavior is a complex interplay between several factors: the nature of the fibers used, surface treatments applied, the matrix selected, and the manufacturing protocols followed [15]. In terms of durability, flax fibers, inherently hydrophilic due to their cellulosic content, introduce additional moisture absorption capabilities, enhancing the overall water uptake of the composites [38]. In contrast, carbon fibers, typically known for their hydrophobic nature, may have less impact on moisture absorption, yet could contribute to variations in composite behavior due to their unique surface chemistries [39]. Water and moisture affect the mechanical, chemical, and physical properties of composites as well as dimensional stability [40,41].

The presence of moisture in these composites has multiple effects. The first is the plasticizing effect, which often occurs in the polymer matrix. This has the effect of reducing chain entanglement when absorbed moisture or water is present, hence boosting chain mobility [42]. The strength and rigidity are consequently reduced [43]. A different effect might happen because of the flax fibers. Water absorption in flax fiber-reinforced composites may cause fibers to swell, increasing stress at the fiber/matrix interface and possibly accelerating microcracking and compromising the mechanical integrity of the composites [18,44]. The carbon fibers, while less susceptible to moisture, may still undergo subtle surface chemistry changes when exposed to prolonged wet environments, possibly affecting the overall structural performance.

Recent advances in the development of flax-carbon hybrid composites have shown promising results regarding their thermal properties and durability. Cheng et al. [45] piloted an investigation into the environmental durability of carbon and flax fiber composites with a polypropylene matrix. Their composites were put to the test under hygrothermal conditions, revealing carbon fibers’ efficacy in moisture reduction and the provision of a protective layer against hygrothermal aging. Unfortunately, the aging process detrimentally affected the tensile and bending strength, particularly in pure flax composites, unveiling a problematic interface between layers susceptible to damage. Similarly, Xian et al. [46] dissected both the impact resistance and the aging attributes of flax/carbon hybrid composite plates bound with epoxy resin. Confirmation that carbon fibers amplified hydrothermal resistance and structural integrity was forthcoming, while the presence of flax fabric notably enhanced the materials’ toughness.

The study conducted by Wong et al. [47] dove further into the nuances of moisture absorption and its effect on tensile behaviors, finding a significant disparity in moisture uptake based on the positioning of flax and carbon layers in the composite–flax on the outside led to a significantly higher rate of moisture absorption (470%). This moisture absorption was determined after submerging the composites in distilled water at 60 °C until saturation.

In a particularly insightful work, Wang et al. [48] interrogated the role of carbon nanofibers in flax fiber-reinforced epoxy’s response to hygrothermal aging. Their examination identified a sweet spot at 1.0 wt.% concentration of CNFs, which dramatically limited water absorption and provided a significant advantage to tensile strength, pointing towards a pathway for notable long-term durability enhancements.

Adding to this body of work, Ramesh et al.’s research [49] provided a comprehensive look at the morphological shifts and physical responses of hybrid flax and carbon fiber-reinforced laminates under hygrothermal stress. Their findings suggested carbon fibers’ inclusion as an effective dampener for water intake, directly correlating fiber distribution with strength and elasticity. Specifically, the water intake characteristics showed that the flax fibers absorbed 92%, carbon fibers absorbed 36.6%, and flax-carbon fiber reinforced composites absorbed 61.9% of water.

Contributing further to the understanding of these materials’ performance, Xian et al. [50] shed light on the hydrothermal durability of unidirectional flax/carbon hybrid composite plates, emphasizing how varying temperatures during water immersion profoundly affect the polymer composites’ structural performance. Once more, the beneficial role of carbon fiber content in reducing water uptake was underscored, emphasizing the strategic layering of fibers for optimizing composite strength.

These studies all come to the same conclusion that flax-carbon hybrid composites have great potential for improving their thermal properties and durability. Together, they explain the intricate relationship between the arrangement of the fibers, the composition of the matrix, and the challenges posed by the environment. As a result, they contribute to the ongoing discussion on how to effectively utilize the combined benefits of hybrid composite materials.

Building on this foundation, the objective of this paper is to expand upon the research path established in the previous studies of authors [51,52] that investigated hybrid flax-carbon fiber polyamide 11 biocomposites in terms of their mechanical characteristics, including tensile, impact, flexural, and hardness properties, as well as their damping capabilities and interlaminar bond strength. Continuing into this realm, exploring thermal properties and durability under environmental challenges is crucial in order to comprehensively characterize these composites. Accomplishing this will not only provide a comprehensive overview of these innovative materials but also support their application in the next generation of advanced engineering solutions where performance and sustainability intersect. In this regard, this study investigates the thermal properties of woven flax-carbon hybrid polyamide biocomposites through DSC, FTIR, and TGA analysis. The durability of these biocomposites is also assessed through water and moisture absorption tests. Furthermore, the degradation and estimated lifetime of both the matrix and the manufactured composites were compared at different operating temperatures.

## 2. Experimental Procedure

### 2.1. Materials

Two types of woven fabrics were utilized in this study with the same pattern of 2 × 2 twill and an areal density of 200 g/m^2^: a flax fabric supplied by Lucio J & M (Madrid, Spain) and a carbon fabric supplied by Castro Composites S.L. (Pontevedra, Spain). Prior to manufacturing the composite materials, both fabrics were kept in storage at room temperature (roughly 23 °C) and relative humidity (approximately 33%). Commercial bio-based PA11, provided by Arkema (Madrid, Spain), was utilized as the thermoplastic matrix. Using a hot-press machine, the PA11 pellets were transformed into sheets in accordance with the process outlined by the authors in their previous work [53].

### 2.2. Surface Treatment

An atmospheric pressure plasma torch (APPT) (Plasma Treat GmbH, Steinhagen, Germany) was employed on the flax fabric surface to address the weak bond identified in earlier studies [54,55,56] to improve the adhesion of flax fabrics with the PA11 matrix. APPT was selected for its capacity to alter the physicochemical characteristics of material surfaces while preserving their bulk properties, offering a rapid, eco-friendly, non-hazardous, and dry modification technique [30,57,58]. Furthermore, APPT presents specific benefits for materials that pose challenges in terms of modification, thereby rendering it ideal for improving bonding between natural fibers and polymer matrices [59,60,61]. It is worth remembering that surface activity and plasma effects tend to decline over time [53]. As a result, the fabric treatment was carried out about 30 min before the composite manufacturing process. In order to preserve the improved adhesion properties of the treated surface and reduce the possibility of its effectiveness declining, a comparatively short gap was chosen. Previous studies [30,62] provided technical specifications and device setup details. While carbon fibers showed good adhesion with the PA11 matrix, no surface treatment was required.

### 2.3. Composite Manufacturing

Laminated composite materials were fabricated employing a thermal compression technique with a hot press (FontijnePresses TPB374, Barendrecht, The Netherlands), following an 80-min processing cycle. The press settings, specifically a final temperature of 200 °C, a pressure of 3 MPa, and a cooling rate of 1.7 °C/min were carefully chosen to align with the thermal behaviors and flow characteristics of both the matrix and the fibers. The 80-min processing cycle plays a vital role in determining the ultimate properties of laminated composite materials. This prolonged cycle facilitates efficient heat transfer, ensuring a uniform distribution of temperature and an effective flow of the matrix. Moreover, it promotes the dispersion of polymer chains, which, in turn, improves the bonding between the matrix and the reinforcing fibers, ultimately enhancing the overall strength of the material. Furthermore, the gradual cooling process helps to reduce residual stresses and prevent thermal distortion, thereby preserving the dimensional stability of the composite. The cycle is precisely optimized to achieve a consistent microstructure, minimizing voids and promoting thorough impregnation of resin, leading to uniform material properties and heightened reliability.

**Figure 1:**
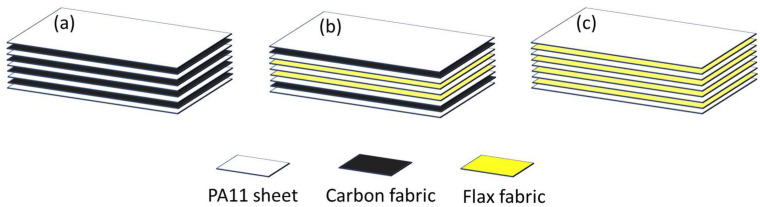
Layup configurations: (**a**) non-hybrid carbon fiber composite ([C_2_]_S_), (**b**) hybrid flax-carbon composite ([CF]_S_), and (**c**) non-hybrid flax fiber composite ([F_2_]_S_).

The resulting laminates consisted of two homogenous versions with four layers of woven carbon (CCCC: [C_2_]_S_) and woven flax (FFFF: [F_2_]_S_), alongside a hybrid version with two internal layers of woven flax sandwiched by two external layers of carbon (CFFC: [CF]_S_). Sheets of Polyamide 11 (PA11) were placed on the outside of the stack and in between the layers of fabric. Table 2 provides a thorough description and specifications of each composite, which are depicted in Figure 1. As described in previous work [52], a weight-based technique was used to determine the volume fraction and quantify the fiber content in the manufactured composites.

### 2.4. Differential Scanning Calorimetry (DSC)

The thermal characteristics of composites were assessed using DSC 822 Mettler Toledo (Greifensee, Switzerland) to identify any possible endothermic or exothermic phase changes, as they were heated in a controlled manner. The experiment was performed using 8–12 mg samples that were heated at a rate of 10 °C/min from 25 °C to 200 °C in an aluminum crucible with a 40 µL capacity. As a purge gas, nitrogen was fed in at a rate of 50 mL/min. The thermal stability, melting point, enthalpy, and degree of crystallinity were all studied by DSC analysis.

The degree of crystallinity was estimated using Equation (1) [63]:

(1) $$X_{C}=\frac{∆H_{m}}{{∆H}_{m^{{}^{\circ}}}\times w}\times 100\%$$

where X_c_ is the degree of crystallinity, ΔH_m_ is the melting enthalpy of the composite, w is the weight fraction of the matrix, and ΔH_m_^o^ is the melting enthalpy of the 100% crystalline PA11 (244 J/g [64]).

### 2.5. Fourier Transform Infrared Spectroscopy (FTIR)

An infrared spectrometer machine was used to capture the composites’ FTIR spectra (Brucker Optic GmbH, Madrid, Spain). The Bruker Tensor 27 spectrometer, which employed a diamond prism with a resolution of 4 cm^−1^, 32 scans, and an incident radiation angle of 45°, was utilized to record the generated spectra at a depth of about 5–10 µm. ATR, or attenuated total reflection, was performed to analyze the surface chemical alterations (FTIR-ATR). For every composite, three spectra were captured to guarantee consistent outcomes.

### 2.6. Thermogravimetric Analysis

Thermogravimetric analysis (TGA) was used to study the stability of the samples, analyzing the behavior of their weight as a function of temperature, as they were controlled and heated under an atmosphere of gaseous nitrogen. The measurements were carried out under a nitrogen flow of 50 mL/min to determine the degradation temperature from 25 to 500 °C (up to 800 °C for the hybrid one) at a heating rate of 10 °C/min. The equipment used for all TGAs was the TGA Q500 from TA Instruments (New Castle, DE, USA), which incorporates a mass spectrometer, MS Discovery (TA Instruments), allowing the molecular weights of the compounds released during the test and their respective temperatures to be determined. Three tests at different heating rates of 6, 10, and 20 °C/min were conducted for each material sample with an approximate weight of 8–10 mg.

### 2.7. Durability Test

The absorbency of water and moisture in the fabricated composites was primarily influenced by the amalgamation of polar amide groups in the polyamide matrix and potential hydrophilic sites within the flax fibers [65,66]. The polar nature of amide groups is due to the presence of nitrogen and oxygen atoms, which permit the formation of hydrogen bonds with water molecules.

This study involved the absorptive behavior of composite materials to water immersion and a humid atmosphere. Specimen sizes were standardized to 20 × 20 × 4 mm^3^ and then placed in two containers following ASTM D570-98 guidelines [67]. To test water absorption, one set of these samples was placed in distilled water at room temperature, while the other set was kept in a second container with a constant temperature of 23 °C and 50% relative humidity to detect moisture uptake. Composite samples were successively taken after two hours, one, seven, fifteen, and thirty days. They were then carefully dried using absorbent paper to remove any surface moisture and were massed immediately. Water and moisture absorption were determined by the percentage weight increase, M_t_, calculated using the following equation:

(2) $$M_{t}=\frac{W_{t}-W_{0}}{W_{0}}\times 100$$

where W_0_ is the weight of the initial specimen in dry conditions and W_t_ is the weight of the wet specimen at each aging time. For every aging period, three specimens were measured.

The Fickian diffusion model stands out as a fundamental framework for describing the transport of water molecules within composite structures [68]. This model is rooted in the principles of mass diffusion and is widely applied to predict absorption kinetics in polymers and composite materials. The Fickian diffusion model assumes that water absorption is primarily governed by molecular diffusion through the material matrix. Fick’s second law (Equation (3)) describes the rate of change of concentration (M) with respect to time (t) and position (x) in a diffusing system. In one dimension, the equation takes the form:

(3) $$\frac{\partial M}{\partial t}=D\frac{\partial^{2}M}{{\partial x}^{2}}$$

where D is the diffusion coefficient. If we consider a scenario in which the diffusion process has reached a steady state (i.e., there is no change in concentration over time), Fick’s second law simplifies to:

$$\frac{\partial M}{\partial t}=0$$

In this case, the concentration profile did not change with time. Solving Fick’s second law, subject to the initial condition, yields the diffusion coefficient in Equation (4) [69,70]:

(4) $$D=\frac{\pi }{16}h^{2}[\frac{\frac{M_{t}}{M_{\infty }}}{\sqrt{t}}{]}^{2}$$

where M_t_ and M_∞_ represent the mass of water absorbed at time t and the equilibrium mass of water absorbed, and h is the sample’s thickness. This equation represents the rate at which water or moisture diffuses into the material.

In addition to the durability evaluation, specimens underwent equivalent aging durations for DSC and FTIR investigations, which provided insight into the structural and chemical changes in the composites caused by the absorption of water and moisture.

### 2.8. Thermal Lifetime Estimation

The study of decomposition reaction kinetics in composite materials was conducted using TGA, as the heating rate of the sample directly impacted the kinetics of its reactions. By employing STAR^e^ software (V12.10, Mettler Toledo GmbH, Greifensee, Switzerland) and analyzing the thermograms obtained for each heating rate, it was feasible to compute the decomposition energy (E_d_) relative to the material’s degradation degree (α), and subsequently estimate the thermal service life of materials. This calculation was facilitated by the kinetic model incorporated in the software, known as Model Free Kinetics (MFK) V12.10 [71], enabling a comparison with the results obtained using the Kissinger model [72].

In the Kissinger model, the activation energy was determined from the temperature corresponding to the peak of the decomposition curve, which varied with the heating rate, as described by Equation (5). This energy is inherent in the slope value obtained from linear fits

(5) $$\ln\left(\frac{\beta }{T_{P}^{2}}\right)=\ln A-\frac{E_{a}}{RT_{P}}$$

where β is the heating rate, T_p_ is the peak temperature (K), R is the gas constant (8.314 J/mol.K), A is a constant, and E_a_ is the activation energy (J/mol) to be calculated. The activation energy by the Kissinger method can be determined by plotting $\ln\left(\frac{\beta }{T_{P}^{2}}\right)$ against 1000/T at different heating rates from Equation (5).

Subsequently, by applying the equation proposed by Toop [73] (Equation (6)), it was possible to calculate the service life of each material as a function of temperature [74]:

(6) $$\ln t_{f}=\frac{E_{a}}{RT_{f}}+\ln\left(\frac{E_{a}}{\beta R}.P\left(X_{f}\right)\right)$$

where β is the heating rate, R is the gas constant, E_a_ is the activation energy calculated for the degree of decomposition (obtained through Kissinger) considered as material failure, in this case 5%, T_f_ is the selected service temperatures for the service life calculation, P(X_f_) is equal to the exponential factor of E_a_/(RT_c_) (T_c_ is the temperature for 5% loss) according to Toop’s theory [73], and finally, t_f_ is the estimated time to material failure. This was compared to the simulation provided by MFK for isothermal decomposition at different temperatures.

## 3. Results

### 3.1. DSC Analysis

Thermal properties were assessed using DSC for all composites. Table 3 presents the measured parameters, including melting temperature (T_m_), recrystallization temperature (T_c_), melting enthalpy (∆H), and degree of crystallinity (X_c_) calculated using Equation (1).

The glass transition temperature (T_g_) was primarily influenced by the polymer matrix and its molecular structure. Therefore, T_g_ values for all composites fell within a similar range. However, the T_g_ values of the composites were lower than those of the pure matrix (PA11) reported in previous work (44.49 °C) [53]. This reduction may be attributed to two factors. Firstly, the addition of reinforcement fibers can have a plasticizing effect, disrupting the polymer chains and facilitating the transition from a glassy to a rubbery state at a lower temperature. Secondly, the incorporation of rigid fillers, such as carbon fibers, can limit polymer chain mobility, resulting in reduced T_g_, as the polymer chains are less able to move freely. This lower T_g_ can result in higher ductility and resistance to the impact of the composites compared to the pure matrix.

The hybrid composite displayed the highest enthalpy value compared to the reference composites. This observation can be attributed to both improved crystallinity and enhanced interfacial bonding. The hybrid composite potentially exhibited enhanced crystallinity owing to the combined presence of flax fibers (contributing to crystalline cellulose) and carbon fibers. The synergistic interaction between these fibers may foster a more ordered arrangement of polymer chains, thereby resulting in higher enthalpy values during the melting process. Furthermore, the hybrid composite may demonstrate superior interfacial bonding between the flax and carbon fibers and the polymer matrix. This enhanced bonding, which was proven in previous work of the authors [51], could lead to a more homogeneous structure, influencing the heat flow during the melting process and contributing to higher enthalpy values.

The increased crystallinity observed in the hybrid composite, along with the enhanced interfacial adhesion between the carbon and flax fibers and the polymer matrix, can potentially affect the mechanical properties by facilitating more effective stress transmission and resistance to deformation. Prior works [51,52] have illustrated enhancements in the mechanical performance of hybrid composites. In particular, these investigations have indicated that the absorbed impact energy, failure strain (ductility), and damping characteristics of hybrid composites exceeded those of the reference carbon composite.

### 3.2. FTIR Analysis

**Figure 2:**
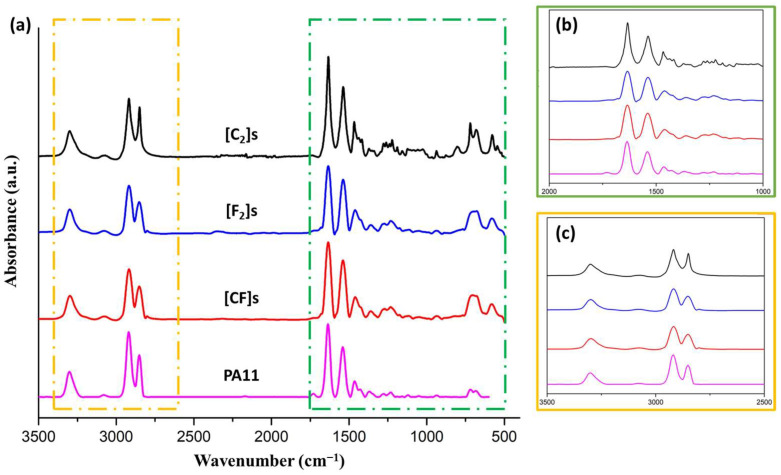
(**a**) FTIR spectra of the matrix, pure flax, pure carbon, and hybrid composites, with magnified spectra in the wavenumber ranges of (**b**) 2000–1000 cm^−1^ and (**c**) 3500–2500 cm^−1^.

Due to the limited depth of FTIR analysis, the obtained spectra for the laminated composites (Figure 2) showed very similar peaks to the PA11 matrix. The characteristic bands for PA11, such as amide I, amide II, and amide III, were observed at specific wavenumbers, along with other characteristic bands for C-H stretching vibrations and N-H stretching. Additionally, a small peak corresponding to the carboxyl group was observed, providing information about possible acid hydrolysis in the material. These results for the PA11 matrix, as reported in Table 4, from our previous study [53], provide additional context for the observed similarity in the spectra of the laminated composites, suggesting that the detected groups in the composites are predominantly representative of the PA11 matrix rather than the individual fiber components. This insight underscores the limitations of the FTIR technique in detecting bulk information and helps provide a more accurate analysis of composite materials. The restricted identification of fibers in the matrix through FTIR assessment can be primarily ascribed to the limited depth of penetration of the FTIR method (5–10 µm), which predominantly analyzes surface layers. Additionally, the arrangement of layers utilized in laminated composites, in which the matrix layer is situated on top, worsens this limitation by confining the assessment to the surface layers.

### 3.3. TGA Analysis

**Figure 3:**
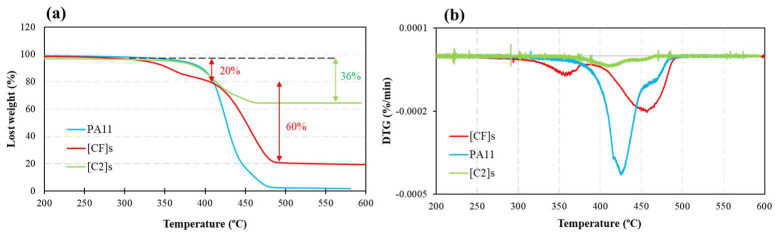
(**a**) TGA and (**b**) DTG curves for PA11, carbon, and hybrid composites at a 10 °C/min heating rate.

Figure 3 depicts the TGA and differential thermogravimetry (DTG) thermograms obtained at a heating rate of 10 °C/min for PA11, carbon, and hybrid composites within a temperature range of 200–600 °C. These curves depict the weight change and its derivative regarding the temperature. A summary of the obtained decomposition temperature peaks (T_d_) for the materials is provided in Table 5, facilitating a comparative analysis of their thermal degradation behaviors.

A parallel decomposition profile was discerned between PA11 and the carbon composite, characterized by a single peak in the DTG curve with comparable T_d_ values (426–431 °C). This similarity highlights the limited impact of carbon fibers on the degradation pathway of PA11, which is attributable to the exceptional thermal stability of carbon fibers, allowing them to endure high temperatures without substantial degradation. While PA11 showed a loss of nearly 100% of its weight at 450 °C, the carbon composite only experienced a 36% weight loss.

Conversely, the hybrid composite revealed a different DTG response, presenting two distinct degradation peaks at 350 °C and 463 °C, attributed to the degradation of hemicelluloses and alpha-cellulose in flax fibers [75,76], and PA11 decomposition, respectively. The observed shift of T_d_ of the matrix from 426 °C for pure PA11 to 431 °C and 463 °C for carbon (1.2% increase) and hybrid composites (8.7% increase), respectively, indicates that while the presence of flax fibers had a negative effect on the onset decomposition temperature of the composites, it contributed to thermally stabilizing the composite once degradation started. The literature suggests comparable enhancements in thermal stability, as observed in studies involving the reinforcement of PA11 with cellulose nanofibers [77], other thermoplastic matrices reinforced with lignocellulosic fibers [78], and PA11-based composites with stone groundwood fibers [79]. This phenomenon has been interpreted as the residue generated from fiber decomposition inhibiting the diffusion of volatile and radical compounds involved in the decomposition of PA11 [79].

The inflection point in the TGA curve (between the first and second peaks), as well as the overlapped peaks observed in the DTG curve for the hybrid composite, indicate that there was a range of temperatures in which both the fibers and matrix degraded simultaneously.

According to Figure 3a, all materials exhibited considerable thermal stability until a high temperature of 310 °C. The residual weights of 64% and 20% for carbon and hybrid composites, respectively, compared to the 100% degradation of the pure matrix after reaching 500 °C, indicate the increased thermal stability of the composites. This enhanced stability can be attributed to the reduced volume fraction of the matrix in the composite resulting from the presence of fibers that have better thermal resistance compared to the polymeric matrix.

The decomposition energies (E_d_) for PA11, carbon, and hybrid composites were evaluated using the Kissinger model, as summarized in Table 6.

**Figure 4:**
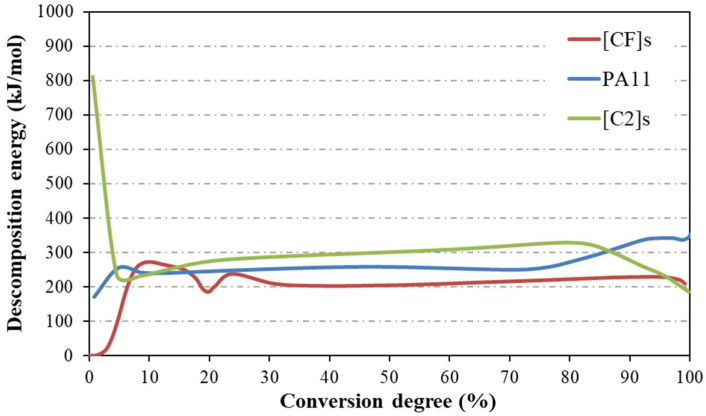
Decomposition energy as a function of the degree of conversion obtained from the MFK method for the materials.

Figure 4 exhibits E_d_ profiles as a function of the degree of conversion, calculated using the MFK method for the studied materials. The data underscored that while the Kissinger model confirmed predictive efficiency at mid-decomposition (around 50%), the MFK model displayed superior precision in the initial decomposition stages, revealing the polymorphic nature of these reactions.

Interestingly, initial decomposition exposed disparate behaviors that eventually stabilized beyond the 20–25% reaction progression threshold. The carbon composite required a noticeable energy of 800 kJ/mol for the onset of decomposition, a phenomenon attributed to the high thermal endurance of carbon fibers combined with polymer impregnation, necessitating greater energy for the commencement of degradation. The maximum E_d_ value, which belonged to the carbon composite, occurred within the 25–80% conversion range, reflecting the outstanding thermal stability inherent in the carbon composite.

After 80% conversion, the PA11 matrix experienced an increase in E_d_ potentially attributable to alterations in the material’s structure or bonding as degradation progressed. Conversely, a decrement in E_d_ was observed for the carbon composite, suggesting changes in material cohesion. Such end-stage E_d_ fluctuations may imply the degree of interfacial adhesion within composites, with better adhesion correlating to increased E_d_. It is crucial to acknowledge that these implications originate from empirical methods, thus necessitating cautious interpretation regarding discrepancies between different materials.

**Figure 5:**
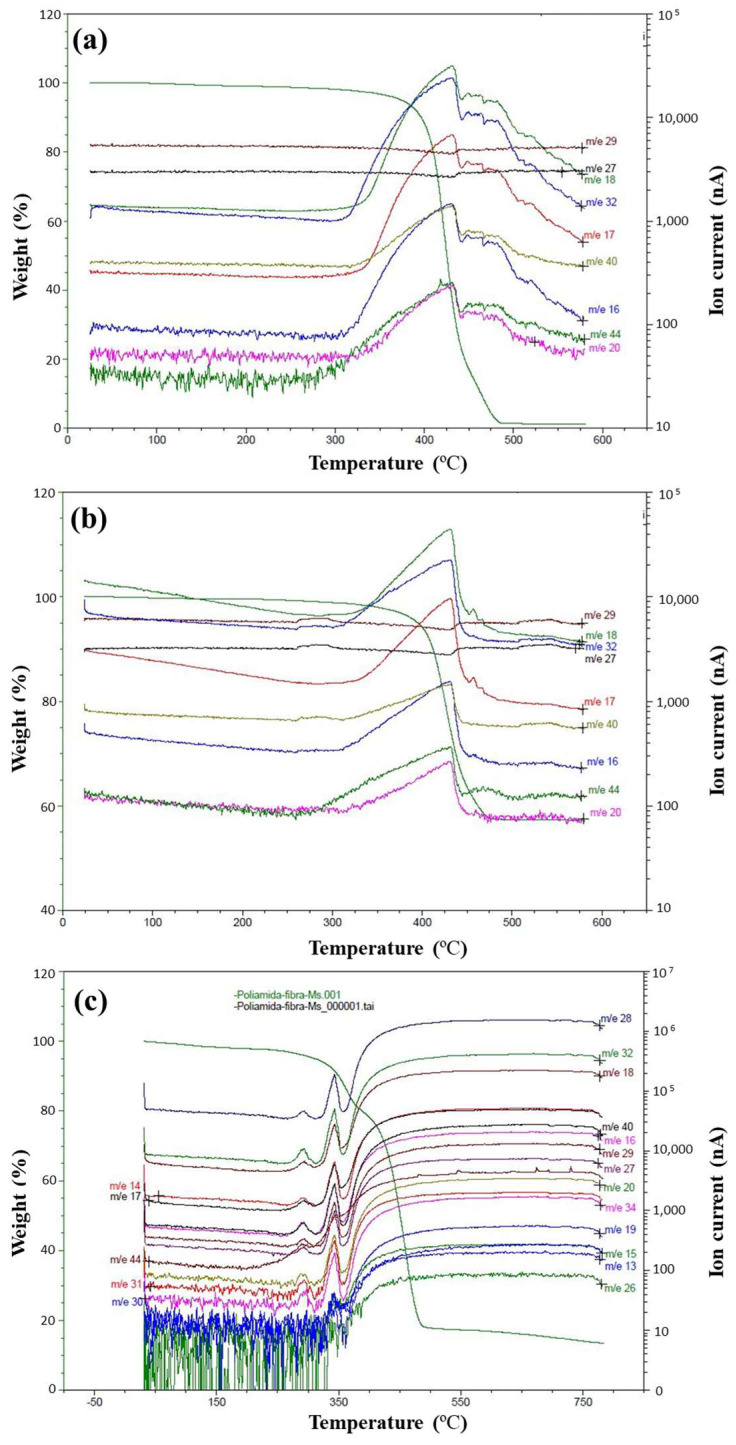
MS thermograms: (**a**) PA11, (**b**) [C_2_]_S_ composite, and (**c**) [CF]_S_ composite.

Simultaneously, mass spectroscopy (MS) analysis was conducted to identify the gases generated during the decomposition process under an inert nitrogen atmosphere, as shown in Figure 5.

The main thermogram peak for PA11 and the carbon composite ranged approximately between 300 °C and 450 °C, reaching a maximum of 430 °C. Interestingly, a minor band for PA11, observed from 450 °C to 500 °C in both Figure 3b and the MS data, could point to the decomposition stage of more stable PA11 components or by-products such as residual monomer or oligomers, which require higher temperatures for volatilization.

Predominantly, the gases released included H_2_O, O_2_, OH^–^, and CH_4_, with slightly variable amounts between PA11 and composites, as detailed in Table 7 with corresponding temperatures. The detection of H_2_O and OH^–^ is typical, as these species commonly emanate from the degradation of organic constituents. Methane’s presence arises from carbon-rich fragments within the materials. Notably, CO detection in hybrid composites is consistent with the degradation of flax fiber’s hemicelluloses and alpha-cellulose content. Moreover, in the hybrid composite, gases from smaller fragments of the chains, such as -CH-, -CH_2_-, or non-stoichiometric nitrogen oxides, were also observed. The charge-mass ratios of 26, 27, and 29 were not considered in Table 7, as they correspond to isotopes of nitrogen gas, which constitutes the atmosphere in which the reaction took place.

### 3.4. Durability Test

**Figure 6:**
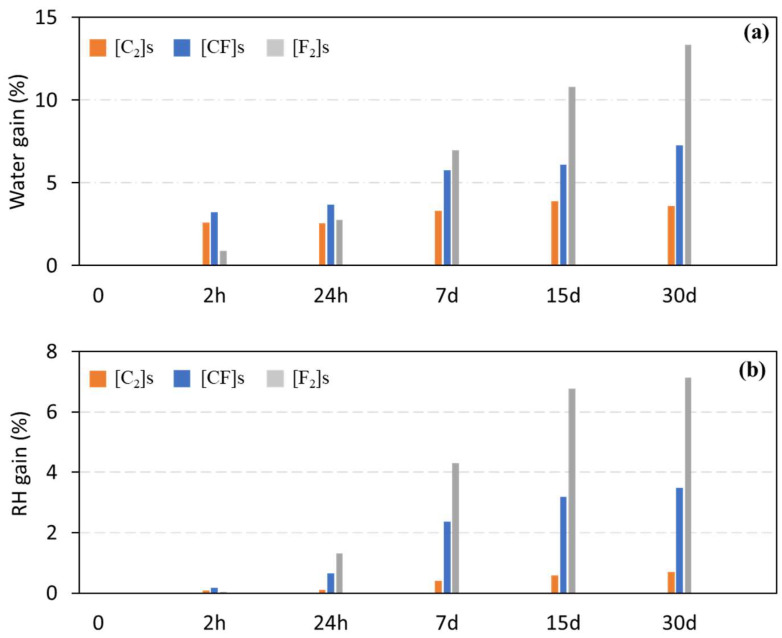
Durability test results over aging periods: (**a**) water gain and (**b**) moisture gain.

The purpose of the durability tests was to study the water and moisture absorption behavior of the manufactured composites. Water and moisture absorption were measured using a relative weight increase (M_t_) across various aging times, in accordance with the standards. Figure 6 depicts the trend, demonstrating an increase in water/moisture gain in all composites over the 30-day aging period. After saturation was reached, the composites’ masses remained stable, indicating a zero-diffusion flow. Additionally, DSC and FTIR measurements were employed to evaluate the composites’ structural and chemical changes due to water immersion and relative humidity exposure.

The carbon composite displayed remarkable resistance to water absorption (Figure 6a), with a minimal absorption of 3.6% over 30 days. In contrast, the flax composite had a significant tendency to absorb water, with a value of 13.3%, which was 3.6 times higher over the same period. Flax fibers’ intrinsic capillary water absorption capacity was the cause of this significant rise. The hybrid composite displayed an intermediate performance, with water absorption measurements beginning close to the carbon composite but ending at a reduced 7.2%.

In contrast to water absorption, the moisture gain after 30 days was considerably lower (Figure 6b), but followed the same trend among the composites. The carbon, flax, and hybrid composites yielded moisture uptakes of 0.7%, 7.1%, and 3.5%, respectively.

The high level of water and moisture absorption in pure flax composites makes them inappropriate for moisture-sensitive applications, like marine environments or outdoor structures exposed to weathering. However, carbon and hybrid composites, characterized by notably reduced absorption rates and enhanced durability, are widely utilized in sectors such as aerospace, automotive, and the manufacturing of sports equipment.

Regarding the Fickian diffusion model, it is only applicable for the moisture absorption results since during the water absorption test, the specimens did not reach the saturation level according to Figure 6a. When plotting the ratio M_t_/M_∞_ against the square root of time, the linearity of the curve in its initial portion serves as a validity criterion for the applicability of Fick’s law. The diffusion coefficient (D) values obtained from the application of the Fickian model are reported in Table 8. These D values provide crucial information about the rate of moisture absorption in the different composite materials. Specifically, the carbon composite exhibited lower diffusivity, indicating a slower diffusion rate of water vapor into the material. This lower diffusion coefficient suggests that the carbon composite is less permeable to moisture and shows a comparatively slower absorption process. In contrast, the flax composite demonstrated the highest D value, suggesting a faster absorptive process due to the high flax capacity of capillary water absorption. Moreover, the hybrid composite displayed an intermediate D value. This intermediate diffusivity reflects a moderated diffusion rate, influenced by the combined characteristics of flax and carbon fibers within the composite.

The differences between water and moisture absorption results primarily stem from the phase of the medium–liquid versus vapor–and the variant pathways and interaction rates with the composite material [64]. Water, in its liquid form, offers a broad interface for absorption, resulting in higher weight gains. Moisture, such as vapor, interacts at a more gradual pace, typically resulting in lower absorption levels [80,81]. Moreover, water vapor molecules have higher kinetic energy than liquid water molecules. This elevated energy allows water vapor to diffuse more rapidly through the material. However, the overall process can be protracted due to the more tortuous diffusion pathway that vapor molecules follow–penetrating smaller pores and gaps within the material’s structure.

Previous research by Bourmaud et al. [82] and Le Duigo et al. [83] supports these findings, highlighting the flax composite’s maximum absorption due to its hydrophilic nature, which may cause components like pectin to dissolve upon immersion. This dissolution process, occurring at the fiber/matrix interface, creates voids that could evolve into microcracks, increasing the number of absorption sites. Conversely, the carbon composite exhibited the least amount of absorption, likely a consequence of the carbon fibers’ hydrophobic properties, which reduces water/moisture penetration. The hybrid composite showed intermediate absorption levels, suggesting that, while the flax component absorbed water/moisture, the effect was somewhat counteracted by the presence of carbon fibers.

After comparing the manufactured composites with the pure polyamide matrix, as described in the authors’ previous study [53], significant differences in the percentage of water/moisture absorption were observed after 30 days of exposure. The pure PA bulk exhibited minimal water and moisture absorption of 1.5% and 0.8%, respectively, at saturation levels. The incorporation of reinforcement fibers into the neat matrix, as also reported in other studies [84,85,86,87], can increase the absorption capacity due to the hydrophilic nature of certain types of fibers, such as flax. Additionally, the interfaces formed between the fibers and the matrix could create regions of increased porosity or interfacial defects. These regions may be more susceptible to water infiltration, allowing water to penetrate deeper into the composite compared to the homogeneous structure of the pure matrix material.

#### 3.4.1. Post-Durability Test DSC Analysis

Table 9 displays the measured thermal characteristics at each aging time. Reflecting the findings from Figure 6, the slight water and moisture absorbance of the carbon composite confirmed the stability of the DSC parameters noted in Table 3, supporting the durability of carbon composites against water-induced structural changes.

Conversely, the high tendency of flax fibers to absorb the water was reflected in the glass transition temperature (T_g_) of the flax composite, which decreased by approximately 8% over 30 days. The decrease in T_g_ suggests that water molecules act as a plasticizer, thereby contributing to a reduction in the polymer chain entanglements and decrease the potential sites for inter-polyamide chain hydrogen bonding. For moisture absorption, the change in T_g_ was marginal (under 1%) due to the earlier attainment of saturation and the lower percentage of moisture absorbed compared to water. The melting temperature (T_m_) had minimal variation, suggesting that the crystalline structures were stable and not significantly influenced by the addition of water or moisture. The rise in enthalpy was observed across the aging timeframe, along with a 3.6% increase in crystallinity after water absorption and a 10.2% increase following moisture exposure. The crystallinity increases can be attributed to two possible effects, on the one hand, to the plasticizing effect of absorbed water molecules, which results in increased chain mobility allowing polymer segments to reorganize into ordered crystalline regions, consequently increasing the enthalpy and crystallinity. On the other hand, lignin, an amorphous flax component, and cellulose, a crystalline constituent, respond to water/moisture unevenly. Hydrolysis of lignin, breaking it down into smaller units, effectively diminishes the amorphous component in flax fibers, thereby relatively enriching the proportion of the crystalline phase in the composite. Thus, the overall growth of fiber crystallinity was observed after water/moisture exposure, contributing to the crystallinity of the flax composite.

The hybrid composite exhibited a discernible trend wherein T_g_ decreased by approximately 17% over 30 days due to water uptake, possibly reflecting an increment in the amorphous phase from the matrix or a weakening in the stiffening effect attributed to the carbon-flax interface. However, for moisture absorption, T_g_ showed a slight increase, likely reflective of the reversible water absorption-desorption behavior [88] associated with materials containing hygroscopic components such as PA11, where further aging beyond saturation could prompt irregular water release, therefore increasing T_g_ mildly. The melting point stayed within a narrow range of 194 °C, confirming the stability of the crystalline parts once more. The observed decreases in enthalpy by 8% and 30% for water and moisture gains, respectively, alongside a corresponding reduction in crystallinity, might suggest progressive structural changes due to prolonged water/moisture incorporation.

While the hybrid composite displayed the highest initial enthalpy according to DSC results (Table 3), it implies improved interfacial bonding and higher crystallinity–indicative of a robust structure less susceptible to water absorption effects. Considering the fluctuation in the T_m_ for the hybrid composite from 193 °C to 199 °C after 30 days in the moisture test, this can be attributed to the complex interplay of the absorption process and the material’s response. A 3.5% absorbed moisture of the hybrid composite might facilitate additional cross-linking or other reactions leading to denser crystalline regions, theoretically increasing T_m_.

#### 3.4.2. Post-Durability Test FTIR Analysis

**Figure 7:**
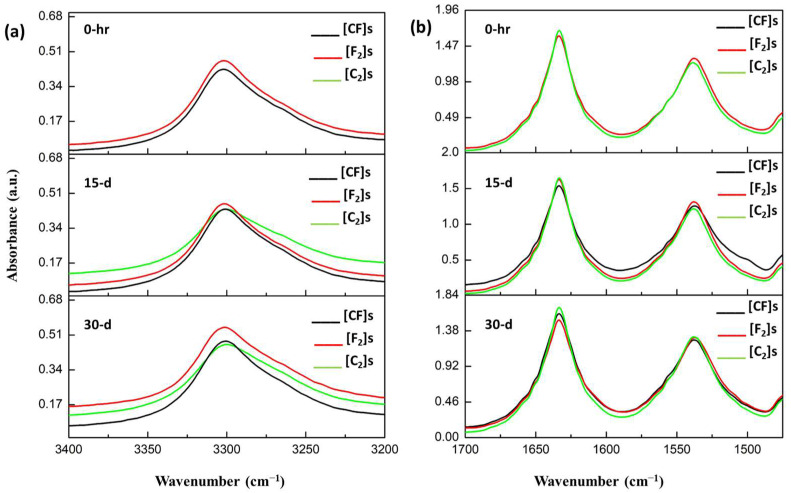
Comparative FTIR spectra post-water absorption: (**a**) OH/NH bands and (**b**) amide I band analysis.

**Figure 8:**
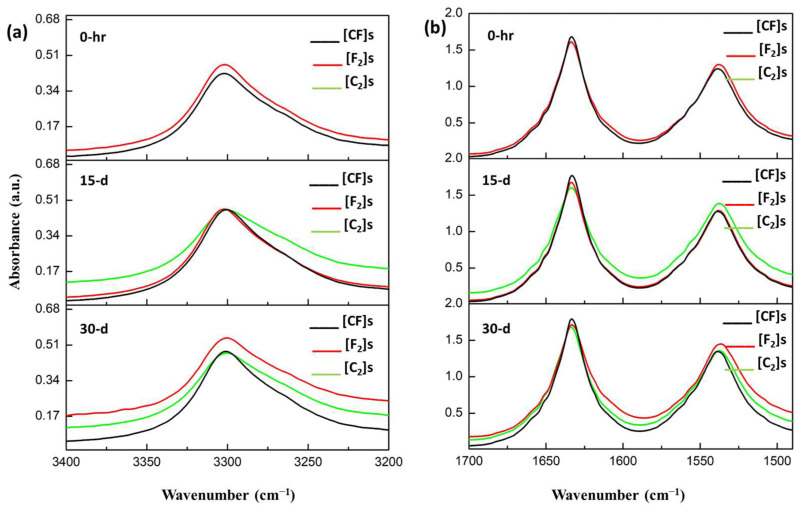
Comparative FTIR spectra post-moisture absorption: (**a**) OH/NH bands and (**b**) amide I band analysis.

Upon completion of a 30-day durability test assessing water/moisture absorption in PA11-based laminate composites, FTIR spectroscopy was employed to investigate changes in the chemical structure of the laminates. Figure 7 and Figure 8 depict the spectra, comparing the bands associated with O-H/N-H stretching (3500–3100 cm^−1^) and amide I deformations and stretches (1750–1500 cm^−1^) after immersion durations.

The FTIR spectra of the composites indicated remarkable consistency throughout the aging period, with minimal observable changes. This is mostly due to the inherent constraint of FTIR in evaluating bulk material properties. Thus, it effectively reflected the surface matrix behavior regarding water/moisture interaction due to its topmost PA11 layer. This observation provides significant evidence that the degradation mechanisms are primarily physical, such as the plasticization of the polymeric matrix, as no noticeable chemical degradation was detected through FTIR analysis.

The only difference, which was also negligible, was a less than 5% increase in the intensity of the O-H/N-H band in the flax composite compared to the others (for both the water and moisture spectra). This suggests that plasma-treated flax fibers engage more actively with absorbed water molecules, limiting their interaction with the PA11 matrix. In other words, activated flax fibers may preferentially bind water molecules due to the relatively higher concentration of accessible OH and NH groups on the fiber surface compared to those available on the PA11 matrix.

### 3.5. Thermal Lifetime Estimation

The STAR program allowed for an isothermal estimation of the service life based on the E_d_ and degree of conversion, as presented in Table 10. To estimate the useful life of all studied materials, it is necessary to define which decomposition degree is considered for material failure. The most common is to consider the failure as soon as the decomposition begins, so it is generally considered at 5% of the decomposition degree [74,89,90,91,92].

Obviously, increasing the temperature decreases the time it would take for the sample to completely degrade. It was observed that the PA matrix displayed a longer time to reach 5% decomposition at any temperature compared to the composites. This difference can be attributed to the decrease in the amount of resin, since as seen in the carbon fiber composite, there is a 60% weight residue and 16% in the hybrid. However, the normalized value of the enthalpy of the decomposition reaction is related to the total weight of the composite sample, and all calculations are related to this enthalpy.

**Figure 9:**
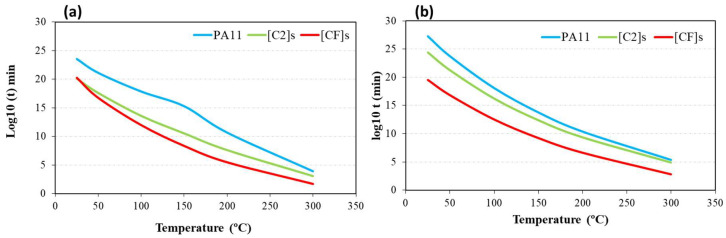
Thermal lifetime estimation: (**a**) MFK method and (**b**) Kissinger and Toop methods for PA11 and its composites.

Figure 9 compares the result of the thermal lifetime simulation carried out with MFk and a combination of Kissinger and Toop methods (for a 5% conversion degree). The values are quite similar, although there are small differences inert to the empirical methods used. Kissinger and Toop’s methods seemed more conservative, giving slightly higher values in all cases. The trends are maintained; regardless of the method used, it can be observed that the hybrid composite presents the lowest time in most of the cases; therefore, its degradation occurs in comparatively shorter times. However, around room temperature, the hybrid composite lifetime was 41% higher than the carbon composite (1.7 × 10^20^ vs. 1.2 × 10^20^ min, MFK method). The carbon composite had a higher matrix volume fraction compared to the hybrid composite, with a 16% difference between the two. Polymer matrices were more susceptible to environmental degradation than fibers at room temperature. Consequently, a lower matrix volume fraction in the hybrid composite may have reduced the material’s overall susceptibility to degradation, contributing to its longer service life at room temperature.

## 4. Conclusions

This study examined the thermal properties, degradation kinetics, and water/moisture absorption behavior of woven flax-carbon hybrid polyamide biocomposites in comparison to non-hybrid composites of pure flax and pure carbon with a PA11 matrix. The main findings were as follows:

- DSC analysis exhibited a higher melting enthalpy (∆H) of the hybrid composite than pure flax or carbon composites, indicating improved crystallinity and potentially better mechanical properties due to a more ordered arrangement of polymer chains.
- FTIR analysis revealed that the spectra of the laminated composites closely resembled those of the PA11 matrix, suggesting that detected groups primarily represent the PA11 matrix rather than individual fiber components.
- TGA analysis revealed remarkable thermal stability of the hybrid composite, comparable to the carbon composite, up to 300 °C without any mass loss. This enhanced thermal stability led to a 41% longer estimated service life of the hybrid composite at room temperature compared to the carbon composite, as determined by the MFK model.
- Durability testing showed that the hybrid composite had about two times lower water/moisture absorption than the flax composite, suggesting superior performance in high water/humidity environments, although carbon composites still exhibited outstanding water/moisture resistance.
- The thermal lifetime simulation results from MFK and a combination of the Kissinger and Toop methods exhibited a similar trend, with the Kissinger and Toop approaches being more conservative, resulting in slightly higher values for service lives.

Overall, the hybrid composites demonstrated a complex interplay of properties resulting from the introduction of flax and carbon fibers into the PA11 matrix. With synergistic benefits from both fiber types, these hybrid materials offered a balance between thermal stability and durability, making them promising for applications requiring a compromise between pure flax and carbon fiber reinforcement in a PA11 matrix while also addressing sustainability and eco-friendliness concepts simultaneously.

## Figures and Tables

**Table 1:** Water absorption comparison of some natural fiber composites.

Natural Fiber Composites	Water Absorption (%)	Reference
Sisal/polyester	7–22	[16,17]
Flax/bio-epoxy	6–12	[18,19]
Jute/polyester	8–20	[20,21]
Hemp/polyester	10–16	[22,23]
Coir/epoxy	2–15	[24,25]

**Table 2:** Properties of the produced laminated composites.

Layup	Average Thickness (mm)	Fiber Volume Fraction (%)		
		Flax V_f_	Carbon V_c_	V_f_ + V_c_
[C_2_]_S_	1.60 ± 0.06	0	30	30
[CF]_S_	2.06 ± 0.03	30	10	40
[F_2_]_S_	2.32 ± 0.04	42	0	42

**Table 3:** Thermal properties of studied polyamides by DSC (values have ±2 error margin).

	[C_2_]_S_	[CF]_S_	[F_2_]_S_
T_g_ (°C)	40.5	39.3	39.2
T_m_ (°C)	193.5	193.5	193.7
∆H (J/g)	23.9	36.1	33.3
X_c_ (%)	16.7	29.7	28.0

**Table 4:** Assignment of the main absorption bands in the FTIR Spectra of PA11 [53].

Frequency (cm^−1^)	Vibration
3301	N-H stretching strong band/OH
3082	NH groups weak band
2918	CH_2_ asymmetric stretching
2850	CH_2_ symmetric stretching
1732	O-C=O
1635	Amide I, C=O stretching
1550	Amide II, C- stretching + C=O in-plane bending
1465	CH_2_ bending asym
1367	CH_2_ bending sym
1275	Amide III, NH-O stretching
1226	C-O-C streching sym/CH_2_ bending
1111	CH_3_ rocking
934	C-C(O) stretching mode (amide IV)
721	CH_2_ rocking/C=O deformation
678	NH out-of-plane mode (amide V)

**Table 5:** Summary of T_d_ values for PA11 and composite materials.

Materials	PA11	[C_2_]_S_	[CF]_S_
T_d_ (°C)	426 ± 8	431 ± 8	350 ± 6
			463 ± 8

**Table 6:** Decomposition energies derived from the Kissinger model.

Materials	PA11	[C_2_]_S_	[CF]_S_
E_d_ (kJ/mol)	290.3	288.5	268.0

**Table 7:** Gases released and corresponding temperatures obtained via mass spectrometry during the degradation process.

Materials	PM (g/mol)	16	17	18	32	44	28	40
	Gas	CH_4_	OH^−^	H_2_O	O_2_	CO_2_	CO	-CH-CH-CH_2_
PA11	Temperature (°C)	430	430	430	432	430	-	430
[C_2_]_S_		430	430	430	432	430	-	430
[CF]_S_		264 310	264 310	264 310	264 310	264 310	264 310	264 310

**Table 8:** Diffusion coefficients for each composite obtained in the durability test.

	D × 10^−8^ (m^2^/s)
	Moisture Absorption
[C_2_]_S_	1.66
[CF]_S_	1.86
[F_2_]_S_	1.94

**Table 9:** Thermal properties of the studied composites post-durability test, as determined by DSC (values have ±2 error margin).

Property	Sample	0 h	2 h	24 h	7 d	15 d	30 d
T_g_ (°C)	[C_2_]_S_ –W ^1^	40	40	38	31	38	32
T_m_ (°C)		193	194	193	194	193	194
∆H (J/g)		24	25	26	25	29	31
χ_c_ (%)		17	17	18	17	20	21
T_g_ (°C)	[CF]_S_ –W	39	41	37	31	32	32
T_m_ (°C)		193	194	195	195	191	194
∆H (J/g)		36	34	28	28	32	33
χ_c_ (%)		29	27	23	23	24	27
T_g_ (°C)	[F_2_]_S_ –W	39	39	38	37	36	36
T_m_ (°C)		193	194	194	192	192	193
∆H (J/g)		33	32	33	34	40	34
χ_c_ (%)		28	27	25	28	33	29
T_g_ (°C	[C_2_]_S_ –RH ^2^	40	39	40	40	38	39
T_m_ (°C)		193	197	192	195	195	192
∆H (J/g)		23	21	29	22	28	24
χ_c_ (%)		16	14	20	15	19	17
T_g_ (°C	[CF]_S_ –RH	39	40	40	40	39	40
T_m_ (°C)		193	195	196	194	197	199
∆H (J/g)		36	33	27	29	24	25
χ_c_ (%)		29	27	22	24	19	20
T_g_ (°C	[F_2_]_S_ –RH	39	39	41	39	40	39
T_m_ (°C)		193	194	194	192	192	193
∆H (J/g)		33	36	33	36	36	36
χ_c_ (%)		28	30	27	30	29	30

^1^ Water; ^2^ Relative humidity.

**Table 10:** Isothermal estimation of lifetime at 5% analysis from MFK.

Materials	(α)	Thermal Lifetime Estimation (Minute)					
		25 °C	50 °C	100 °C	150 °C	200 °C	300 °C
PA11	5%	3.5 × 10^23^	1.3 × 10^21^	6.9 × 10^17^	1.9 × 10^15^	4.5 × 10^10^	7255.1
[C_2_]_S_		1.2 × 10^20^	3.7 × 10^17^	3.6 × 10^13^	3.2 × 10^10^	3.8 × 10^7^	1089.8
[CF]_S_		1.7 × 10^20^	5.2 × 10^16^	8.9 × 10^11^	2.1 × 10^8^	2.9 × 10^5^	47.1

## Data Availability

The datasets generated during and/or analyzed during the current study are available from the corresponding author upon reasonable request.

## References

[1] Al-Oqla F.M., Sapuan S., Anwer T., Jawaid M., Hoque  M. (2015). Natural fiber reinforced conductive polymer composites as functional materials: A review. Synth. Met..

[2] Rohan T., Tushar B., GT M. (2018). Review of natural fiber composites. Proc. IOP Conf. Ser. Mater. Sci. Eng..

[3] More A.P. (2022). Flax fiber–based polymer composites: A review. Adv. Compos. Hybrid Mater..

[4] Baley C., Gomina M., Breard J., Bourmaud A., Davies P. (2020). Variability of mechanical properties of flax fibres for composite reinforcement. A review. Ind. Crops Prod..

[5] Amroune S., Belaadi A., Bourchak M., Makhlouf A., Satha H. (2022). Statistical and experimental analysis of the mechanical properties of flax fibers. J. Nat. Fibers.

[6] Le Duigou A., Davies P., Baley C. (2011). Environmental impact analysis of the production of flax fibres to be used as composite material reinforcement. J. Biobased Mater. Bioenergy.

[7] Dissanayake N.P., Summerscales J., Grove S., Singh M. (2009). Energy use in the production of flax fiber for the reinforcement of composites. J. Nat. Fibers.

[8] Foulk J., Akin D., Dodd R., Ulven C. (2011). Production of flax fibers for biocomposites. Cellulose Fibers: Bio- and Nano-Polymer Composites—Green Chemistry and Technology.

[9] Deng Y., Guo Y., Wu P., Ingarao G. (2019). Optimal design of flax fiber reinforced polymer composite as a lightweight component for automobiles from a life cycle assessment perspective. J. Ind. Ecol..

[10] Sekar S., Suresh Kumar S., Vigneshwaran S., Velmurugan G. (2022). Evaluation of mechanical and water absorption behavior of natural fiber-reinforced hybrid biocomposites. J. Nat. Fibers.

[11] Gholampour A., Ozbakkaloglu T. (2020). A review of natural fiber composites: Properties, modification and processing techniques, characterization, applications. J. Mater. Sci..

[12] Karimah A., Ridho M.R., Munawar S.S., Adi D.S., Damayanti R., Subiyanto B., Fatriasari W., Fudholi A. (2021). A review on natural fibers for development of eco-friendly bio-composite: Characteristics, and utilizations. J. Mater. Res. Technol..

[13] Asim M., Paridah M.T., Chandrasekar M., Shahroze R.M., Jawaid M., Nasir M., Siakeng R. (2020). Thermal stability of natural fibers and their polymer composites. Iran. Polym. J..

[14] Joseph P., Joseph K., Thomas S., Pillai C., Prasad V., Groeninckx G., Sarkissova M. (2003). The thermal and crystallisation studies of short sisal fibre reinforced polypropylene composites. Compos. Part A Appl. Sci. Manuf..

[15] Neto J.S., de Queiroz H.F., Aguiar R.A., Banea M.D. (2021). A review on the thermal characterisation of natural and hybrid fiber composites. Polymers.

[16] Kumari Y.R., Ramanaiah K., Prasad A.R., Reddy K.H., Sanaka S.P., Prudhvi A.K. (2021). Experimental investigation of water absorption behaviour of sisal fiber reinforced polyester and sisal fiber reinforced poly lactic acid composites. Mater. Today: Proc..

[17] Sreekumar P., Thomas S.P., marc Saiter J., Joseph K., Unnikrishnan G., Thomas S. (2009). Effect of fiber surface modification on the mechanical and water absorption characteristics of sisal/polyester composites fabricated by resin transfer molding. Compos. Part A Appl. Sci. Manuf..

[18] Muñoz E., García-Manrique J.A. (2015). Water absorption behaviour and its effect on the mechanical properties of flax fibre reinforced bioepoxy composites. Int. J. Polym. Sci..

[19] Moudood A., Rahman A., Khanlou H.M., Hall W., Öchsner A., Francucci G. (2019). Environmental effects on the durability and the mechanical performance of flax fiber/bio-epoxy composites. Compos. Part B Eng..

[20] Akil H.M., Cheng L.W., Ishak Z.M., Bakar A.A., Abd Rahman M. (2009). Water absorption study on pultruded jute fibre reinforced unsaturated polyester composites. Compos. Sci. Technol..

[21] Dash B., Rana A., Mishra H., Nayak S., Tripathy S. (2000). Novel low-cost jute–polyester composites. III. Weathering and thermal behavior. J. Appl. Polym. Sci..

[22] Dhakal H.N., Zhang Z.a., Richardson M.O. (2007). Effect of water absorption on the mechanical properties of hemp fibre reinforced unsaturated polyester composites. Compos. Sci. Technol..

[23] Shahzad A. (2012). Effects of water absorption on mechanical properties of hemp fiber composites. Polym. Compos..

[24] Das G., Biswas S. (2016). Effect of fiber parameters on physical, mechanical and water absorption behaviour of coir fiber–epoxy composites. J. Reinf. Plast. Compos..

[25] Mittal M., Chaudharya R. (2019). Effect of Fiber Length and Content on Mechanical and Water Absorption Behavior of Coir Fiber-Epoxy Composite. Advanced Engineering Research and Applications.

[26] Prem Kumar R., Muthukrishnan M., Felix Sahayaraj A. (2023). Effect of hybridization on natural fiber reinforced polymer composite materials—A review. Polym. Compos..

[27] Gangil B., Ranakoti L., Verma S., Singh T., Kumar S. (2020). Natural and synthetic fibers for hybrid composites. Hybrid Fiber Composites: Materials, Manufacturing, Process Engineering.

[28] Gupta M., Ramesh M., Thomas S. (2021). Effect of hybridization on properties of natural and synthetic fiber-reinforced polymer composites (2001–2020): A review. Polym. Compos..

[29] Bahrami M., Abenojar J., Martínez M.Á. (2020). Recent progress in hybrid biocomposites: Mechanical properties, water absorption, and flame retardancy. Materials.

[30] Bahrami M., Enciso B., Gaifami C.M., Abenojar J., Martinez M.A. (2021). Characterization of hybrid biocomposite Poly-Butyl-Succinate/Carbon fibers/Flax fibers. Compos. Part B Eng..

[31] Yuan W., Li Y., Zhao J. (2021). Mechanical properties of a novel Tri-directional carbon-flax-aramid fiber reinforced composite. Compos. Sci. Technol..

[32] Biricik G.D., Celebi H., Seyhan A.T., Ates F. (2022). Thermal and mechanical properties of flax char/carbon fiber reinforced polyamide 66 hybrid composites. Polym. Compos..

[33] Dora K.N., Mishra S.S., Gupta H.D., Sahu D., Srivatsava M., Dalai N. (2022). Enhancement of surface & bulk mechanical properties of Flax reinforced composites with different carbon allotropes. Mater. Today Proc..

[34] Bahrami M., Mehdikhani M., Swolfs Y., Abenojar J., Martínez M.A. Impact properties of flax-carbon hybrid composites under low-velocity impact. Proceedings of the 20th European Conference on Composite Materials (ECCM20).

[35] Yashas Gowda T.G., Vinod A., Madhu P., Mavinkere Rangappa S., Siengchin S., Jawaid M. (2022). Mechanical and thermal properties of flax/carbon/kevlar based epoxy hybrid composites. Polym. Compos..

[36] Saha A., Kumar S., Zindani D. (2021). Investigation of the effect of water absorption on thermomechanical and viscoelastic properties of flax-hemp-reinforced hybrid composite. Polym. Compos..

[37] El-Wazery M., El-Kelity A., Elsad R. (2020). Effect of water absorption on the tensile characteristics of natural/synthetic fabrics reinforced hybrid composites. Int. J. Eng..

[38] Moudood A., Rahman A., Öchsner A., Islam M., Francucci G. (2019). Flax fiber and its composites: An overview of water and moisture absorption impact on their performance. J. Reinf. Plast. Compos..

[39] Tanaka K., Mizuno S., Honda H., Katayama T., Enoki S. (2013). Effect of water absorption on the mechanical properties of carbon fiber/polyamide composites. J. Solid Mech. Mater. Eng..

[40] Nguyen P.H., Spoljaric S., Seppälä J. (2018). Redefining polyamide property profiles via renewable long-chain aliphatic segments: Towards impact resistance and low water absorption. Eur. Polym. J..

[41] Pérez-Pacheco E., Cauich-Cupul J., Valadez-González A., Herrera-Franco P. (2013). Effect of moisture absorption on the mechanical behavior of carbon fiber/epoxy matrix composites. J. Mater. Sci..

[42] Meyer A., Jones N., Lin Y., Kranbuehl D. (2002). Characterizing and modeling the hydrolysis of polyamide-11 in a pH 7 water environment. Macromolecules.

[43] Paolucci F., Peters G.W., Govaert L.E. (2020). Plasticity-controlled failure of sintered and molded polyamide 12: Influence of temperature and water absorption. J. Appl. Polym. Sci..

[44] Lu M.M., Fuentes C.A., Van Vuure A.W. (2022). Moisture sorption and swelling of flax fibre and flax fibre composites. Compos. Part B Eng..

[45] Cheng M., Zhong Y., Kureemun U., Cao D., Hu H., Lee H.P., Li S. (2020). Environmental durability of carbon/flax fiber hybrid composites. Compos. Struct..

[46] Wang A., Wang X., Xian G. (2020). Mechanical, low-velocity impact, and hydrothermal aging properties of flax/carbon hybrid composite plates. Polym. Test..

[47] Johar M., Chong W., Wong K. (2023). Moisture Absorption and Tensile Behaviour of Hybrid Carbon/Flax Composites. Fibers Polym..

[48] Wang Y., Zhu W., Wan B., Meng Z., Han B. (2021). Hygrothermal ageing behavior and mechanism of carbon nanofibers modified flax fiber-reinforced epoxy laminates. Compos. Part A Appl. Sci. Manuf..

[49] Ramesh M., Bhoopathi R., Deepa C., Sasikala G. (2018). Experimental investigation on morphological, physical and shear properties of hybrid composite laminates reinforced with flax and carbon fibers. J. Chin. Adv. Mater. Soc..

[50] Wang A., Liu X., Yue Q., Xian G. (2023). Hydrothermal durability of unidirectional flax/carbon fiber hybrid composite plates. J. Mater. Res. Technol..

[51] Bahrami M., Del Real J.C., Mehdikhani M., Butenegro J.A., Abenojar J., Martínez M.Á. (2023). Hybridization effect on interlaminar bond strength, flexural properties, and hardness of carbon–flax fiber thermoplastic bio-composites. Polymers.

[52] Bahrami M., Butenegro J.A., Mehdikhani M., Swolfs Y., Abenojar J., Martinez M.A. (2023). Tensile, impact, and the damping performance of woven flax-carbon hybrid polyamide biocomposites. Polym. Compos..

[53] Bahrami M., Abenojar J., Martínez M.A. (2021). Comparative characterization of hot-pressed polyamide 11 and 12: Mechanical, thermal and durability properties. Polymers.

[54] Kumar D., Faisal N., Layek A., Priyadarshi G. (2021). Enhancement of mechanical properties of carbon and flax fibre hybrid composites for engineering applications. AIP Conf. Proc..

[55] Enciso B., Abenojar J.a., Martínez M. (2017). Influence of plasma treatment on the adhesion between a polymeric matrix and natural fibres. Cellulose.

[56] Enciso B., Abenojar J., Paz E., Martínez M. (2018). Influence of low pressure plasma treatment on the durability of thermoplastic composites LDPE-flax/coconut under thermal and humidity conditions. Fibers Polym..

[57] Tiwari S., Bijwe J. (2014). Surface treatment of carbon fibers-a review. Procedia Technol..

[58] Conrads H., Schmidt M. (2000). Plasma generation and plasma sources. Plasma Sources Sci. Technol..

[59] Jang B.Z. (1992). Control of interfacial adhesion in continuous carbon and Kevlar fiber reinforced polymer composites. Compos. Sci. Technol..

[60] Sánchez M.L., Patino W., Cardenas J. (2020). Physical-mechanical properties of bamboo fibers-reinforced biocomposites: Influence of surface treatment of fibers. J. Build. Eng..

[61] Gleissner C., Landsiedel J., Bechtold T., Pham T. (2022). Surface activation of high performance polymer fibers: A review. Polym. Rev..

[62] Bahrami M., Butenegro J.A., Abenojar J., Martinez M.A. (2024). Adhesion characteristics of plasma-treated flax fabrics and elastoplastic properties of their biocomposites. J. Adhes..

[63] Campoy I., Gomez M., Marco C. (1998). Structure and thermal properties of blends of nylon 6 and a liquid crystal copolyester. Polymer.

[64] Wunderlich B. (2005). Thermal Analysis of Polymeric Materials.

[65] Acierno S., Van Puyvelde P. (2005). Rheological behavior of polyamide 11 with varying initial moisture content. J. Appl. Polym. Sci..

[66] Huner U. (2015). Effect of water absorption on the mechanical properties of flax fiber reinforced epoxy composites. Adv. Sci. Technol. Res. J..

[67] (1985). Standard Test Method for Water Absorption of Plastics.

[68] Collings T. (1994). Moisture absorption–Fickian diffusion kinetics and moisture profiles. Handbook of Polymer Fibre Composites UK: Longman Scientific and Technical.

[69] Correlo V.M., Pinho E.D., Pashkuleva I., Bhattacharya M., Neves N.M., Reis R.L. (2007). Water absorption and degradation characteristics of chitosan-based polyesters and hydroxyapatite composites. Macromol. Biosci..

[70] Abot J., Yasmin A., Daniel I. (2005). Hygroscopic behavior of woven fabric carbon-epoxy composites. J. Reinf. Plast. Compos..

[71] Vyazovkin S., Wight C.A. (1999). Model-free and model-fitting approaches to kinetic analysis of isothermal and nonisothermal data. Thermochim. Acta.

[72] Blaine R.L., Kissinger H.E. (2012). Homer Kissinger and the Kissinger equation. Thermochim. Acta.

[73] Toop D.J. (1971). Theory of life testing and use of thermogravimetric analysis to predict the thermal life of wire enamels. IEEE Trans. Electr. Insul..

[74] Florez T.A., Aparicio G.M. (2014). Thermal characterization and lifetime estimation of the humus lombricospt. Am. J. Anal. Chem..

[75] Van de Velde K., Baetens E. (2001). Thermal and mechanical properties of flax fibres as potential composite reinforcement. Macromol. Mater. Eng..

[76] Khalfallah M., Abbès B., Abbès F., Guo Y., Marcel V., Duval A., Vanfleteren F., Rousseau F. (2014). Innovative flax tapes reinforced Acrodur biocomposites: A new alternative for automotive applications. Mater. Des..

[77] Panaitescu D.M., Frone A.N., Nicolae C. (2013). Micro-and nano-mechanical characterization of polyamide 11 and its composites containing cellulose nanofibers. Eur. Polym. J..

[78] Ardanuy M., Antunes M., Velasco J.I. (2012). Vegetable fibres from agricultural residues as thermo-mechanical reinforcement in recycled polypropylene-based green foams. Waste Manag..

[79] Oliver-Ortega H., Méndez J.A., Mutjé P., Tarrés Q., Espinach F.X., Ardanuy M. (2017). Evaluation of thermal and thermomechanical behaviour of bio-based polyamide 11 based composites reinforced with lignocellulosic fibres. Polymers.

[80] Ramesh M., Tamil Selvan M., Niranjana K. (2022). Hygrothermal Aging, Kinetics of Moisture Absorption, Degradation Mechanism and Their Influence on Performance of the Natural Fibre Reinforced Composites. Aging Effects on Natural Fiber-Reinforced Polymer Composites: Durability and Life Prediction.

[81] Jean J.Y., Mallon P.E., Schrader D.M. (2003). Principles and Applications of Positron and Positronium Chemistry.

[82] Bourmaud A., Morvan C., Baley C. (2010). Importance of fiber preparation to optimize the surface and mechanical properties of unitary flax fiber. Ind. Crops Prod..

[83] Le Duigou A., Davies P., Baley C. (2013). Exploring durability of interfaces in flax fibre/epoxy micro-composites. Compos. Part A Appl. Sci. Manuf..

[84] Qian S., Wang H., Zarei E., Sheng K. (2015). Effect of hydrothermal pretreatment on the properties of moso bamboo particles reinforced polyvinyl chloride composites. Compos. Part B Eng..

[85] Masoodi R., Pillai K.M. (2012). A study on moisture absorption and swelling in bio-based jute-epoxy composites. J. Reinf. Plast. Compos..

[86] Daramola O.O., Adediran A.A., Adewuyi B.O., Adewole O. (2017). Mechanical properties and water absorption behaviour of treated pineapple leaf fibre reinforced polyester matrix composites. Leonardo J. Sci..

[87] Hamdan M.H.M., Siregar J.P., Cionita T., Jaafar J., Efriyohadi A., Junid R., Kholil A. (2019). Water absorption behaviour on the mechanical properties of woven hybrid reinforced polyester composites. Int. J. Adv. Manuf. Technol..

[88] Venoor V., Park J.H., Kazmer D.O., Sobkowicz M.J. (2021). Understanding the effect of water in polyamides: A review. Polym. Rev..

[89] Marcovich N.E., Reboredo M.M., Aranguren M.I. (2001). Modified woodflour as thermoset fillers: II. Thermal degradation of woodflours and composites. Thermochim. Acta.

[90] Batista N.L., Costa M.L., Iha K., Botelho E.C. (2015). Thermal degradation and lifetime estimation of poly (ether imide)/carbon fiber composites. J. Thermoplast. Compos. Mater..

[91] Enciso B., Abenojar J., Aparicio G., Martínez M. (2021). Decomposition kinetics and lifetime estimation of natural fiber reinforced composites: Influence of plasma treatment and fiber type. J. Ind. Text..

[92] Paik P., Kar K.K. (2008). Kinetics of thermal degradation and estimation of lifetime for polypropylene particles: Effects of particle size. Polym. Degrad. Stab..

